# Determination of reference values for dynamic spinal parameters using video rasterstereography

**DOI:** 10.1186/1748-7161-8-S2-O33

**Published:** 2013-09-18

**Authors:** Robert Percy Marshall, Philip Catala-Lehnen

**Affiliations:** 1 University Hospital Hamburg-Eppendorf, Department of Sports-Medicine, Hamburg, Germany

## Background

Rasterstereography has been used clinically for static back measurements for more than three decades. In contrast to X-radiation, which was the sole diagnostic tool for imaging spinal deformities for many years, rasterstereography is a radiation-free method of projecting light beams onto the subject’s back. Allowing the precise identification of the 3-dimensional topography of the back’s surface. As a result, an exact match of the underlying spine can be created[1] by using Turner-Smith’s spine model. The original rasterstereography however, provides static parameters: it does not consider patients’ motion and its effects on the spine. Current development has improved rasterstereography devices, enabling dynamic measurements[2] to be taken.

## Purpose

The purpose of this study is to establish reference values for clinically established parameters in static spinal analysis[3] of healthy subjects measured under standardised dynamic conditions. Understanding spinal dynamic motion in healthy subjects will help to find pathological parameters and plan more individual therapy regimes.

## Methods

A total of 100 healthy subjects (50 males and 50 females), aged 20-40 years, were measured using the newly developed dynamic rasterstereography at constant speed. Special questionnaires were created to standardise the cohort: back pain within the past six months, spinal surgery or chronic diseases were exclusion criteria. Additionally, subjects were clinically examined with respect to their spinal movement (Schober’s Test, Ott Test, etc.) to exclude further statistical outliers.

## Results

For the first time, this study gives gender-specific reference values for healthy subjects aged 20-40 years measured with dynamic rasterstereography. Additionally, the authors will compare the raised data with pathological examinations either self-measured or given by corresponding hospitals and research-fellows. This will further prove the system’s validity as well as suggest how the reference values can be used in future therapy regimes, such as to plan and protocol conservative treatment.

## Conclusions and discussion

This study shows that dynamic rasterstereography enables more insight into spinal motion and, thus, helps doctors and therapy personnel to protocol therapy outcomes as well as their initial goals.
